# Introduction of a team mentoring structure in a new academic Division of Hospital Medicine

**DOI:** 10.1002/jhm.70221

**Published:** 2025-11-04

**Authors:** Haruka Torok, Kathleen Lane, Elizabeth Davis, Donna Coetzee, Andrew P. J. Olson

**Affiliations:** ^1^ Division of Hospital Medicine University of Minnesota Minneapolis Minnesota USA

## Abstract

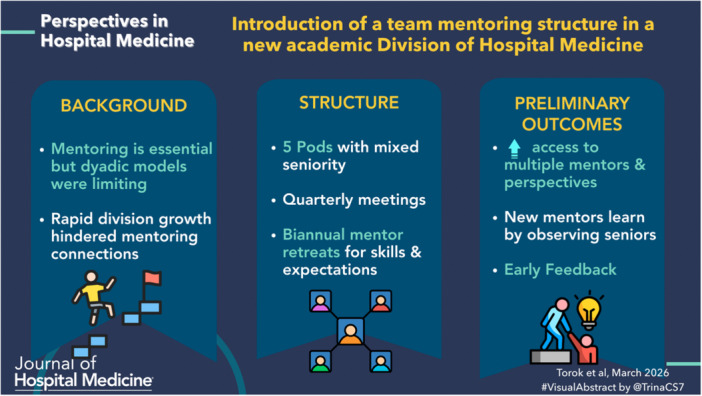

## INTRODUCTION

Strong mentoring is widely recognized as a critical component of successful career development in academic medicine.^1^, ^2^, ^3^ Effective mentoring supports professional identity formation, helps faculty identify areas of interest, facilitates career progression, promotes work‐life integration, fosters retention in academic medicine, and, in turn, cultivates the development of future mentors. However, barriers such as limited time, lack of mentor availability, limited access to experienced mentors, poor‐quality mentoring, and misaligned mentor‐mentee relationships continue to hinder effective mentoring in Hospital Medicine (HM).^3^, ^4^

This article describes the implementation of a novel team‐based mentoring structure within a newly established Division of Hospital Medicine (DHM) at a large academic institution, designed to address several common barriers to effective mentorship.

### Background and needs assessment

The DHM at the University of Minnesota was launched in July 2022, having previously existed as a section within the Division of General Internal Medicine. At the time of its launch, the vast majority of the 60 DHM faculty members were in the rank of assistant professor, with the exception of three associate and one full professor, and devoted most of their time to clinical care.

During the first month, a strategic visioning session was held that was open to all faculty and administrative staff to identify divisional priorities. Key areas that emerged included clinical coaching, mentorship, career development, and divisional support for research and scholarship. While each assistant professor was assigned (or self‐selected) a division mentor as part of a departmental mentoring program, challenges persisted in establishing effective divisional mentorship over the first year. In response, the division gathered senior DHM members—including all division leaders, full and associate professors, and selected experienced assistant professors (approximately 15 faculty)—followed by a division‐wide engagement workshop. These sessions surfaced several mentoring challenges and gaps in DHM, including a shortage of senior faculty relative to the number of junior faculty (DHM faculty are hired at a rank of assistant professor, and we loosely define junior faculty as those who are not in the process of promotion to an associate professor), less experienced faculty assuming mentor roles earlier than typical, limited research mentoring, difficulty identifying opportunities and connecting junior faculty with project leaders, unclear expectations regarding the roles of mentors and mentees, and time constraints stemming from the clinically demanding schedules in both mentor and mentee roles. These barriers align with those described in the literature. We also learned that rapid divisional growth reduced opportunities for forming organic, inclusive mentoring relationships.

### Structure and implementation of the pod mentoring model

In fall 2024, DHM launched a team‐based mentoring model called “Pods.” This structure divided the division into five smaller units, or Pods, each led by a DHM leader and comprising several mentor‐mentee pairs. By this time, the DHM faculty had grown to 76—still primarily assistant professors—but now included 11 associate and 2 full professors, with each Pod consisting of approximately 15 faculty members. The mentor‐mentee dyads were randomly assigned to one of the five pods to make the sizes of each pod similar with the consideration of experienced mentors evenly distributed across the Pods (note that more experienced mentors had more mentees while junior mentors had one or two). We also ensured that various scholarly interests were represented in each pod to expose early‐career faculty—many still exploring their academic niche—to a range of perspectives, opportunities, and career paths.

Each Pod meets quarterly for 1‐h as virtual meetings that replaced one of the existing division‐wide meetings. Administrative staff manages virtual breakout rooms, and each faculty is assigned to their Pod's breakout room upon joining the meeting. This platform was selected to allow workplace flexibility and remove the need of reserving multiple meeting rooms. The mentors in each pod facilitate the meetings using discussion prompts and materials prepared by DHM leadership, while remaining flexible to address topics most relevant to the group. After each meeting, a meeting summary is submitted to the division that includes the topics discussed and the summary of the discussion. Attendance is tracked by administrative staff.

### Mentor training

DHM hosts mentor retreats biannually for all DHM faculty members who have a division mentee. The group includes all of the associate and full professors, division leaders, and assistant professors who are either in the process of promotion or selected as a division mentor by a junior faculty, and the attendance is expected. These forums are 1‐h hybrid workshops on selected themes and led by internal and external experienced mentors. Our first retreat focused on developing division mentor expectations, including meeting frequencies and the role and scope of DHM mentoring. The group determined that the primary focus be career navigation, advising, and sponsoring, with a project collaboration optional. The subsequent retreats focused on skill development for mentoring across differences (i.e., gender differences, underrepresented minority in medicine, cultural diversity, and career trajectories), providing peer‐support for newer mentors within the group, and collaboratively developing mentoring resources.

### Preliminary outcomes

The demographic information of the pod members, meeting themes and attendance are described in Table 1. The initial impact of the Pods was assessed through attendance rates, participant feedback submitted by emails and meeting summaries.

**Table 1:** Characteristics of the pod members and themes and attendance of the first two pod sessions.

Pod (number of faculty in the pod)	Number of division mentors (number of associate or full professors)	Number of female‐identifying faculty (number of mentors)	Discussion themes and number of participants in each pod (number of division mentors in the group)		Number of faculty who participated in at least one session (total 45 out of 75)
			Session 1: Get to know each other “What is your career mission?” “What are you working on or interested in outside of direct patient care?” “What is the best career advice you have ever received?”	Session 2: “What professional network do you have?” “How did you establish them?” “What are the opportunities you are looking for?” Introduce and fill out a Mentor Map	
Pod 1 (16)	4 (2)	6 (1)	6 (2)	5 (2)	9
Pod 2 (15)	3 (3)	7 (1)	7 (3)	4 (1)	9
Pod 3 (15)	5 (2)	6 (2)	6 (3)	5 (2)	8
Pod 4 (14)	5 (2)	7 (2)	6 (3)	5 (3)	9
Pod 5 (15)	4 (3)	10 (2)	7 (3)	8 (3)	10

The review of the meeting summaries showed that many Pods used the discussion prompts, but additionally shared resources and opportunities, and addressed junior faculty's questions. The flexible format allowed Pods to tailor discussions to participants’ needs. For example, one pod that happened to have all female‐identifying attendees at one point explored challenges facing women in medicine. Another Pod group that had a junior faculty member and two senior mentors at the beginning of the session focused the initial part of the session on the junior faculty's career development. One Pod started a shared document detailing members’ scholarly experiences, interests, and challenges so those who could not make the Pod meeting could contribute asynchronously. Additionally, junior mentors reported gaining valuable insights by observing how senior colleagues approached mentoring conversations.

## DISCUSSION

Mentoring in HM typically incorporates several well‐known forms—traditional dyadic, group, and peer mentoring.^2^, ^5^, ^6^, ^7^, ^8^, ^9^ In recent years, there has been growing discussion about the limitations of traditional dyadic mentoring in HM and the importance of implementing structured mentorship programs for junior faculty.^4^, ^8^, ^10^

One strategy successfully implemented by several institutions is group mentoring and peer mentoring structures, typically consisting of several junior faculty members with or without a senior faculty member who facilitates the meetings over time.^2^, ^8^, ^9^ While both pods and group mentoring structures improve access to experienced mentors, the main difference is that the pods include all faculty members regardless of the rank or years of experience with multiple mentors in a group, and address the challenge of less experienced faculty assuming mentor roles by providing valuable opportunities for the new mentors to work alongside experienced mentors while avoiding additional strain on the small number of experienced faculty members.

Mentor training is viewed as essential to effective mentoring. The training structures range from one‐time orientation, lunchtime sessions, didactics, lectures, seminars, workshop, forums, and mentoring for mentors.^11^ Our pod structure is a form of mentoring for mentors, allowing them to mentor together longitudinally and providing opportunities for peer observation. This, in addition to regular retreats, provides more comprehensive training for new mentors.

Encouraging junior faculty to create a panel of mentors and networks is another strategy often used to meet the mentees’ various career needs.^10^ While we strongly emphasize the importance of faculty members building their own mentoring network, we aimed to support this process by providing exposure to multiple mentors within DHM. The pod that encompasses various expertise in a group can mitigate mentor‐mentee misalignment and poor mentoring quality issues while providing more sponsorship opportunities for junior members.

Time constraints and availability may be more challenging to address. However, by converting one of the existing division‐wide meetings to pod format, we increased the chances of junior faculty attending mentoring meetings without adding more to the faculty schedule. Considering ways for the Pod groups to connect asynchronously—such as creating shared documents and meeting minutes—as demonstrated by one of our pods—may need to be explored.

In summary, our Pod model introduced a unique team‐based mentoring strategy, one that is not described elsewhere. This model, where several mentor‐mentee dyads are combined to form a larger group, offers several unique advantages: (1) placing mentor‐mentee dyads within the same Pod enhances their interaction frequency, (2) non‐cohorting by scholarly interest allows “undifferentiated” junior faculty to benefit from exposure to diverse academic trajectories, perspectives and opportunities, (3) each junior faculty gains the opportunity to work with experienced mentors, (4) newer mentors gain developmental opportunities by observing experienced mentors in action, and (5) the structured, virtual format with scheduled sessions improves accountability and accessibility.

Further evaluation is needed to assess the model's impact on faculty engagement, retention, and scholarly productivity. Nonetheless, there is currently a lack of publications detailing innovative and successful approaches to FD in HM,^10^ and our pod model offers a promising, scalable framework for mentoring in large academic HM divisions facing similar challenges.

## CONFLICT OF INTEREST STATEMENT

The authors declare no conflicts of interest.
